# Development of a molecularly imprinted polymer-based electrochemical sensor for the selective detection of nerve agent VX metabolite ethyl methylphosphonic acid in human plasma and urine samples

**DOI:** 10.1007/s00216-024-05155-6

**Published:** 2024-01-25

**Authors:** Sermet Sezigen, S. Irem Kaya, Nurgul K. Bakirhan, Sibel A. Ozkan

**Affiliations:** 1 Department of Medical CBRN Defense, University of Health Sciences, Ankara, Türkiye; 2 Department of Analytical Chemistry, Gulhane Faculty of Pharmacy, University of Health Sciences, Ankara, Türkiye; 3 Department of Analytical Chemistry, Faculty of Pharmacy, Ankara University, Ankara, Türkiye

**Keywords:** VX, Ethyl methyl phosphonic acid, Electrochemical sensor, Molecularly imprinted polymers, Plasma, Urine

## Abstract

This study focuses on the detection of ethyl methyl phosphonic acid (EMPA), a metabolite of the banned organophosphorus nerve agent VX. We developed an electrochemical sensor utilizing the molecularly imprinted polymer (MIP) based on 4-aminobenzoic acid (4-ABA) and tetraethyl orthosilicate for the selective detection of EMPA in human plasma and urine samples. The 4-ABA@EMPA/MIP/GCE sensor was constructed by a thermal polymerization process on a glassy carbon electrode and sensor characterization was performed by cyclic voltammetry and electrochemical impedance spectroscopy. The 4-ABA@EMPA/MIP/GCE sensor demonstrated impressive linear ranges 1.0 × 10^–10^ M–2.5 × 10^–9^ M for the standard solution, 1.0 × 10^–10^ M–2.5 × 10^–9^ M for the urine sample, and 1.0 × 10^–10^ M–1 × 10^–9^ M of EMPA for the plasma sample with outstanding detection limits of 2.75 × 10^−11^ M (standard solution), 2.11 × 10^−11^ M (urine), and 2.36 × 10^−11^ M (plasma). The sensor exhibited excellent recovery percentages ranging from 99.86 to 101.30% in urine samples and 100.62 to 101.08% in plasma samples. These findings underscore the effectiveness of the 4-ABA@EMPA/MIP/GCE as a straightforward, highly sensitive, and selective interface capable of detecting the target analyte EMPA in human plasma and urine samples.

**Supplementary Information:**

The online version contains supplementary material available at 10.1007/s00216-024-05155-6.

## Introduction

**Fig. 1:**
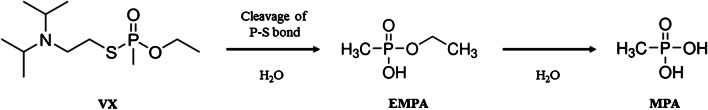
Hydrolysis metabolites of VX

Chemical warfare agents (CWA) are toxic chemicals that cause temporary incapacitation, permanent harm, or death to human beings. Organophosphorus nerve agents (OPNAs) are G-type agents (tabun, sarin, soman, and cyclosarin), V-type agents (VX, RVX, and CVX), and Novichok agents (A 230, A232, and A234). In recent years, OPNAs were used in several chemical terrorist attacks, including the 1994 Matsumoto incident (sarin), 1994 Osaka assassination (VX), 1995 Tokyo incident (sarin), 2013 Damascus incident (sarin), 2017 Kuala Lumpur assassination (VX), 2018 Salisbury assassination and Amesbury incident (Novichok), and 2020 Alexei Navalny assassination (Novichok) [1–5]. After inhalational or cutaneous exposure in humans or animals, absorbed OPNA inhibits the acetylcholinesterase (AChE) enzyme, which controls the hydrolysis of the neurotransmitter acetylcholine. This inhibition causes the accumulation of acetylcholine at the neuromuscular junction that leads to a cholinergic crisis characterized by muscarinic symptoms, including salivation, lacrimation, urination, defecation, gastrointestinal distress, and emesis, known by the mnemonic “SLUDGE” [2, 4, 6, 7]. VX (C_11_H_26_NO_2_PS) is one of the lethal OPNAs, and its median lethal dose (LD_50_) of percutaneous exposure is less than 10 mg for a 70 kg person [5]. After absorption through the skin, the eyes, and the respiratory system, VX mainly accumulates in fatty tissues, leading to delayed systemic distribution, and is metabolized in three forms, including intact compound, degradation products, and protein adducts [4, 8, 9]. One of the VX degradation pathways consists of two steps where VX is hydrolyzed to ethyl methyl phosphonic acid (EMPA) and EMPA is hydrolyzed to methyl phosphonic acid (MPA), respectively (Fig. 1) [3]. MPA is the final hydrolysis metabolite of G and V-type OPNAs excluding tabun. However, there is limited pharmacokinetic data on VX in the literature as VX incidents are rarely seen (two assassinations and one laboratory incident), and only low-exposure VX doses were used in human studies [4, 9, 10].

After a chemical terrorist attack, biological samples, including blood and urine samples of chemical casualties with “SLUDGE” symptoms, should be analyzed to confirm the retrospective verification of the exact type of OPNA exposure for forensic purposes and to manage medical countermeasures [6, 9, 11]. Although blood and urine are the most accessible biological samples early after a chemical terrorist attack where OPNAs were used, collecting the samples from the chemical casualties, labeling them, storing the samples at 4°C, and transferring them in the cold chain to the designated laboratories, needs an efficient sample transport plan using cold chain. Besides, the analysis of biological samples needs a structural organization with complex analytical instruments and an experienced team [6, 11, 12]. In 2023, the Organization for the Prohibition of Chemical Weapons (OPCW) designated a total of 19 laboratories from 14 States Parties for the analysis of authentic biomedical samples in the whole world, so after a chemical attack, a limited number of laboratories could analyze biomedical samples [13]. Besides, a mass casualty situation could worsen all the sampling operations. For this reason, first responders and medical personnel need field deployable point-of-care (POC) devices that can perform highly specific and sensitive detection by using biological samples that could be collected from the chemical casualties with non-invasive or invasive methods. Both blood and urine are ideal biological samples for the real-time monitoring and detection of OPNA exposure [9, 14]. EMPA is an unambiguous biomarker of VX poisoning that could confirm the alleged use of OPNA.

In recent years, efforts have been made toward using molecularly imprinted polymers (MIPs) for constructing electrochemical transducer platforms. Numerous studies have been performed which have aimed to develop new sensors and immobilization schemes based on the integration of innovative materials [15]. MIPs have been employed to improve the sensitivity and selectivity of the designed sensors. These sensors might open up new opportunities for lab-on-a-chip technology. Molecular imprinting is a well-known fabrication technique for designing artificial receptors and molecular sensors [16]. To achieve plasma and urinary EMPA recognition in aqueous solutions, creating MIPs is required to bind the specific target without non-specific binding selectively. A MIP-based electrochemical sensor has been developed in this study with voltammetric techniques due to their unique properties such as low cost, fast response time, environment friendliness, and easy usage [15, 17–19]. Concerning this matter, we aimed to design a MIP-based electrochemical sensor to achieve sensitive, selective, and precise determination of EMPA in plasma and urine samples. The proposed sensor was fabricated by thermal polymerization (TP) process at 50 °C with 4-aminobenzoic acid (4-ABA) monomer in the presence of sodium dodecyl sulfate (SDS) and tetraethyl orthosilicate (TEOS): ethanol. TEOS is an effective matrice to prepare a porous network for MIP structure. The surfactant SDS plays a role in the silane polymerization with TEOS and contributes to the formation of a porous silica network. Thanks to these auxiliary components, polymerization occurs through the interaction between the functional monomer and the template. The polymerization process is based on non-covalent interactions between the functional monomer and the template. Thanks to this interaction, weak bonds are formed through functional groups. During the removal process, these bonds are broken, and the template is removed. 4-ABA@EMPA/MIP/GCE sensor showed sensitive and selective results with EMPA determination from plasma and urine samples. In the literature, there has been no study with selective EMPA detection by electrochemical techniques. This study will illuminate new pathways for researchers studying the verification of OPNA poisonings in chemical casualties of terrorist attacks and shed light on their investigations.

## Materials and methods

### Chemicals

EMPA was purchased from Sigma-Aldrich. Chemical reagents including methanol (MeOH) (99.8%), ethanol (EtOH) (99.8%), acetonitrile (ACN), dopamine, ascorbic acid, uric acid, acetic acid (HAc) (99.0%), hydrochloric acid (HCl), SDS, TEOS, ammonia, isopropyl methyl phosphonic acid (IMPA), n-benzylphosphonic acid (n-BPA), methyl phosphonic acid, 4-ABA (≥99%), commercial human plasma, and synthetic urine were procured from Sigma-Aldrich. Additionally, potassium ferricyanide (99.0%) and potassium ferrocyanide (99.0%) were obtained from Merck. The standard EMPA stock solution (10^−3^ M) was prepared daily in MeOH under the fume hood. A solution containing 5.0 mM [Fe(CN)_6_]^3−/4−^ was prepared in 0.1 M KCl. A glassy carbon electrode (GCE) (3 mm diameter, BASi Technicol, USA) was used as the working electrode. Ag/AgCl (3 M NaCl) and platinum wire serve as the reference electrode and counter electrode, respectively. Electrochemical measurement solutions were prepared using ultrapure water and then stored at 4°C in the refrigerator.

### Development of 4-ABA@EMPA/MIP/GCE sensor via thermal polymerization

The GCE surface should be cleaned and polished before forming MIP film on the surface. For this purpose, a 1:1 (v/v) mixture of MeOH and double distilled water is prepared, and the GCE is incubated in it for 15 min using an ultrasonic bath. Following this, an alumina slurry and a polish pad are used for the second part of the surface cleaning. GCE is rinsed with distilled water and dried at room temperature. First, to prepare the TP solution, 20 μL each was taken from 1.0 mM EMPA stock solution, 1.0 mM 4-ABA solution, 1.0 mM SDS, and 0.1 M NH_3_ solution and they were added to an Eppendorf tube. This solution was mixed using a vortex mixer for 30 s. Twenty microliters of TEOS:EtOH (1:1, v/v) mixture was added to this solution and mixed in a vortex mixer for 90 s. One microliter of this solution was taken and dropped onto the GCE surface. Then, the GCE was kept in a 50 °C oven for 15 min and at room temperature for 5 min. As a result, the 4-ABA@EMPA/MIP/GCE sensor was prepared. In the next stage, non-imprinted polymer (NIP), prepared by following the same processes as MIP but without adding EMPA to the TP solution, is used to control the performance of MIP at each step. The point where NIP differs from MIP is that it does not contain target-specific cavities.

### Commercial human plasma and urine sample preparation

Plasma and urine stock solutions, with EMPA at a concentration of 1 × 10^−3^ M, were prepared separately by adding 1 mL stock solution of EMPA (1 × 10^−2^ M), 3.6 mL plasma/urine, and 5.4 mL of ACN in 12 mL centrifuge tubes. The mixtures of plasma and urine samples with EMPA were centrifugated under 5000 rpm for 10 min. The obtained supernatant of each tube was taken and utilized in electrochemical experiments. Working solutions were created by diluting 5.0 mM [Fe(CN)_6_]^3−/4−^ in 0.1 M KCl. Recovery studies were conducted by spiking a known concentration of pure EMPA solution into a known concentration of the plasma and urine solutions using differential pulse voltammetry (DPV) to assess the EMPA amount in the plasma and urine samples.

### Instrumentation

A PalmSens Potentiostat (Netherlands) was used for all electrochemical measurements, running PSTrace 5.8 software. The electrochemical cell system consisted of a GCE as the working electrode, an Ag/AgCl (3 M KCl) electrode as the reference electrode, and a Pt wire electrode as the counter electrode, establishing a three-electrode configuration. We used a precision balance from Ohaus Instruments (Shanghai, China) for weighing purposes, a vortex mixer manufactured by ISOLAB Laborgeräte GmbH (Eschau, Germany), and an ultrasonic bath from J.P. Selecta (Barcelona, Spain) for solution mixing. Furthermore, for the polymerization and sensor preparation steps, we utilized a Thermo-Shaker (Biosan TS-100), and 4-ABA@EMPA/MIP/GCE sensor was fabricated at 50 °C in a vacuum oven (Jeiotech, OV4-30, Lab Companion, Republic of Korea). All preparations and experiments were performed under the fume hood with an activated carbon filter system (Dorutek, Ankara, Turkiye).

### Scanning electron microscopy (SEM)

Morphological characterizations of the electrode surface were carried out by using Zeiss EVO 40 (Carl Zeiss AG, Oberkochen, Germany) at ×3 and ×10 magnifications.

Fourier transform infrared (FTIR) spectrum in the wave number range of 4000 and 600 cm^−1^ was recorded with the ATR-FTIR 8000 series in Shimadzu, Japan.

## Results and discussion

### Surface characterization of 4-ABA@EMPA/MIP/GCE sensor

**Fig. 2:**
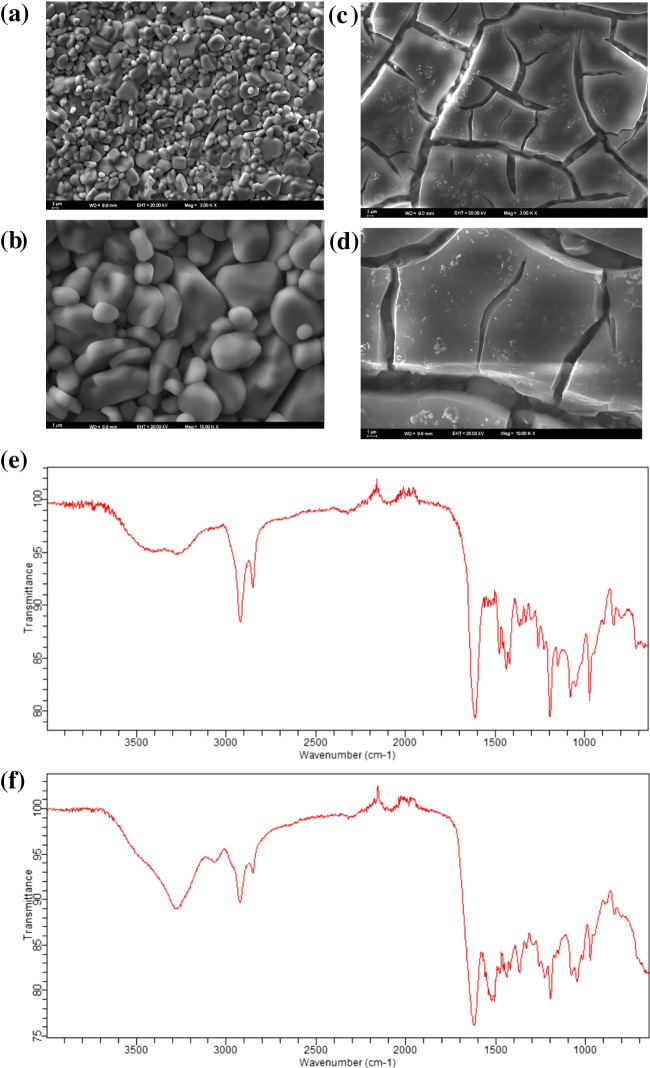
Morphological characterizations of the electrode surface. SEM micrographs of **a**–**b** MIP surface, **c**–**d** NIP surface at ×3 and ×10 magnifications, and **e**–**f** FT-IR spectrums of MIP and NIP, respectively

The molecularly imprinted polymeric film was characterized by using SEM measurements. These analyses were conducted to examine the morphological features of the MIP-based sensor surface and to draw a comparison with the NIP-based surface. According to the obtained results, the SEM images revealed that the MIP surface exhibited roughness and granularity, indicating the presence of a polymer network with readily accessible recognition sites for EMPA. In contrast, the NIP surface appeared smooth [20, 21]. These results can be explained by the specific polymer network within the MIP-based sensor, which is designed to have distinct binding sites for EMPA, resulting in its rough and granular texture (Fig. 2a–d).

To validate the functional groups present in the developed material, a thorough examination of the developed sensor’s chemical composition was conducted. This analysis involved studying the attenuated total reflectance-Fourier transform infrared (ATR-FTIR) spectra within the 4000–650 cm^−1^ wavenumber range. From Fig. 2e–f, the broad *ν*(N–H) band located at around 3280 cm^−1^ and the weak *ν*(C–N) band at around 1332~1300 cm^−1^ come from poly 4-ABA. The two strong bands at 1615 and 1194 cm^−1^ for 4-ABA are assigned to the *ν*(C=O) and *ν*(C–OH) group of carboxylic acid (–COOH). The two weak bands at 1477 and 1420 cm^−1^ are assigned to the asymmetric (–COO^−^as) and the symmetric (–COO^−^s) stretches of the carboxylate group, respectively. These results reveal the poly 4-ABA for the MIP component. The IR peak of 1040 cm^−1^ is assigned to the vibration mode of *ν*_*s*_(C-O-(P)) [22]. The asymmetry mode of *ν*_as_(C-O-(P)) is assigned at 1077 cm^−1^ [23]. The IR frequency at 1149 and 1194 cm^−1^ is assigned to *ν*_as_(C-O-(P)) and *ν*_as_(C-O-(P)), respectively [24]. The IR peaks appearance at 1228 and 1261 cm^−1^ is assigned to *ν*(P = O) adsorbed in the MIP structure, respectively. The IR peak appearance at 1194 cm^−1^ is assigned to *ρ*(P-CH_3_) [25]. The IR frequency at 1315 cm^−1^ is assigned to *δ*_*s*_(CH_3_-P) [26]. The IR frequencies at 1080 and 1051 cm^−1^ are assigned to the overlapped vibration mode of P-Ox and/or PO_2_ by resonance structure forming from P = O and P-O^−^ [27]. The broad absorption peak at 3453 cm^−1^ is assigned to the stretching vibration of structural water -OH. A slightly intense absorption peak at 1623~1615 cm^−1^ is attributed to the bending vibration of O-H due to the trapped water molecules inside the sample. The absorption peaks in both MIP and NIP samples at **~**800 cm^−1^ are attributed to the stretching and bending vibrations of Si-O-Si which belongs to the TEOS compound [28–30]. Furthermore, when comparing MIP to NIP, no discernible distinction was observed, as both polymer types, NIP and MIP, incorporated the identical functional monomer, 4-ABA. This outcome serves as evidence that the functional monomer was effectively integrated into the polymeric frameworks.

### Electrochemical characterization of 4-ABA@EMPA/MIP/GCE

**Fig. 3:**
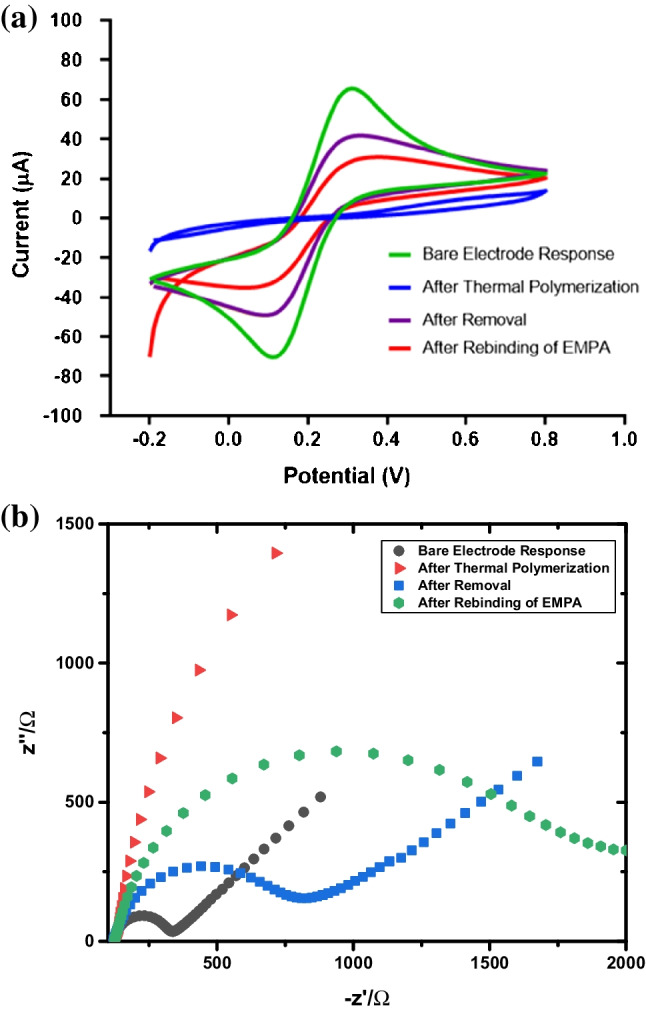
CV voltammograms (**a**) and EIS Nyquist plots (**b**) obtained during the formation of the MIP-based sensor (for CV, potential scan range −0.2 to +0.8 V, scan rate 0.05 V/s, step potential 0.01 V; for EIS, minimum frequency 0.1 Hz, maximum frequency 100,000 Hz, *E*_ac_ 0.01 V)

Electrochemical characterization with cyclic voltammetry (CV) is essential in the creation stages of the 4-ABA@EMPA/MIP/GCE sensor. In all measurements, 5 mM [Fe(CN)_6_]^3−/4−^ solution was utilized as the redox probe, and its electrochemical oxidation peak was observed. Figure 3a shows the obtained CV voltammograms of the redox probe before TP, after TP, after the removal of EMPA, and after the rebinding of EMPA. As a result of easy, fast, and effective electron transfer on the electrode surface, the peak of the redox probe is at the highest current value at bare GCE (before TP). After the TP process, 4-ABA@EMPA/MIP was formed on the surface, and the presence of a polymeric film on the electrode surface resulted in obstruction of electron transfer, which caused the redox probe peak to disappear. As mentioned, target-specific cavities are obtained in the MIP structure thanks to the removal process. These cavities also allow electron transfer to occur, at least partially, on the surface. This causes the peak of the redox probe to increase, although not as much as the bare GCE. After the rebinding of EMPA, the cavities were closed, and electron transfer was prevented again. This is expressed by the decrease in peak current. Another way to electrochemically characterize the 4-ABA@EMPA/MIP/GCE sensor is electrochemical impedance spectroscopy (EIS). In this method, Nyquist plots are utilized to demonstrate the changes in the charge transfer resistance (*R*_ct_) value of the GCE surface. Measurements were taken in the same steps as the CV method (Fig. 3b). However, in the EIS method, the fast and easy electron transfer on the GCE surface is explained by lower *R*_ct_ values. Consequently, the highest *R*_ct_ value belongs to after TP stage, and the lowest belongs to the bare GCE.

### Parameter optimization for TP-assisted MIP process

#### Monomer: template ratio

Optimization steps are critical to obtain an effective and stable result in the MIP preparation process. The first step in this process is to optimize the functional monomer: template ratio. For this purpose, the difference between the peak currents after removal and after TP (Δ*I*_1_) was evaluated versus the functional monomer ratio (1:1, 2:1, 3:1, 4:1, and 5:1). If this ratio is lower than optimum, functional groups cannot be adequately located in the polymer structure, which results in specific cavities not being formed sufficiently. If this ratio is too high, functional monomers will be distributed randomly within the polymer rather than forming specific cavities. The highest Δ*I*_1_ value belongs to the 1:1 ratio, and this ratio was applied in further experiments while preparing the TP solution (Fig. S1a).

#### Dropping volume

After determining the monomer, template ratio to be used when preparing the TP solution and combining it with other components, the next step is to optimize the amount taken from the solution and dropped onto the GCE surface (dropping volume). If the dropping volume is too low or too high, it affects the MIP film thickness and consistency. Here, the effect of volumes ranging from 0.25 to 1.25 μL was observed (Fig. S1b). The highest and optimum value was obtained with increasing values up to 1 μL. For this reason, 1 μL was chosen as the optimum dropping volume. The decrease seen after 1 μL may be related to the MIP film being too thick and removal not occurring.

#### TP temperature and time

Since the TP process is based on heat application, optimizing the TP temperature is very important. For this purpose, the effects of different temperatures varying between 30 and 70 °C were assessed versus the Δ*I*_1_ value. While similar results were obtained at 30, 40, 60, and 70 °C, a significant increase and difference was observed at 50 °C (Fig. S1c). After choosing the optimum temperature, TP time was optimized as the next step. At this step, Δ*I*_1_ values were evaluated versus 5, 10, 15, 20, and 25 min heat application. It was seen that an effective and stable MIP was obtained after 15 min, and it was determined to be the optimum value (Fig. S1d). Increasing both temperature and time beyond the optimum resulted in removal processes being less effective.

#### Removal step

The removal step involves selecting an appropriate solution and time to remove the target molecule and create specific cavities. At this stage, it is necessary to make a careful evaluation because the polymer should not be damaged while removing the template molecule. The solutions whose effects were examined are EtOH, MeOH, 4 M HCl, ACN, and 17.4 M HAc (Fig. S1e). After TP, the electrode was immersed in the selected removal solutions using a ThermoShaker at 650 rpm. After that, it was rinsed with distilled water, and DPV measurements were performed in the redox probe. While EtOH, MeOH, and ACN were almost ineffective in removing EMPA, HAc gave the best and most reproducible results. At the next stage, removal time was optimized using the optimum removal solution between 10 and 30 min. It was seen that, after obtaining the highest Δ*I*_1_ value within 20 min, it decreased and stayed almost the same. Hence, 20 min was selected as the optimum value (Fig. S1f).

#### Rebinding time

Rebinding time, the last step of the optimization procedure, explains the time to incubate 4-ABA@EMPA/MIP/GCE in the requested concentrations of EMPA. In this parameter, which affects the determination and sensor response time, the difference between after removal and after rebinding current values (Δ*I*_2_) was calculated. At this stage, very high binding of the template to specific spaces may result in reaching saturation more quickly. In the study, which was conducted with durations varying between 5 and 20 min, the optimum duration was 5 min (Fig. S1g). Once the sensor is prepared according to the optimized parameters explained above, it can be used repeatedly throughout the day for all measurements without preparing the sensor again.

### Analytical validation and performance evaluation of 4-ABA@EMPA/MIP/GCE sensor

**Fig. 4:**
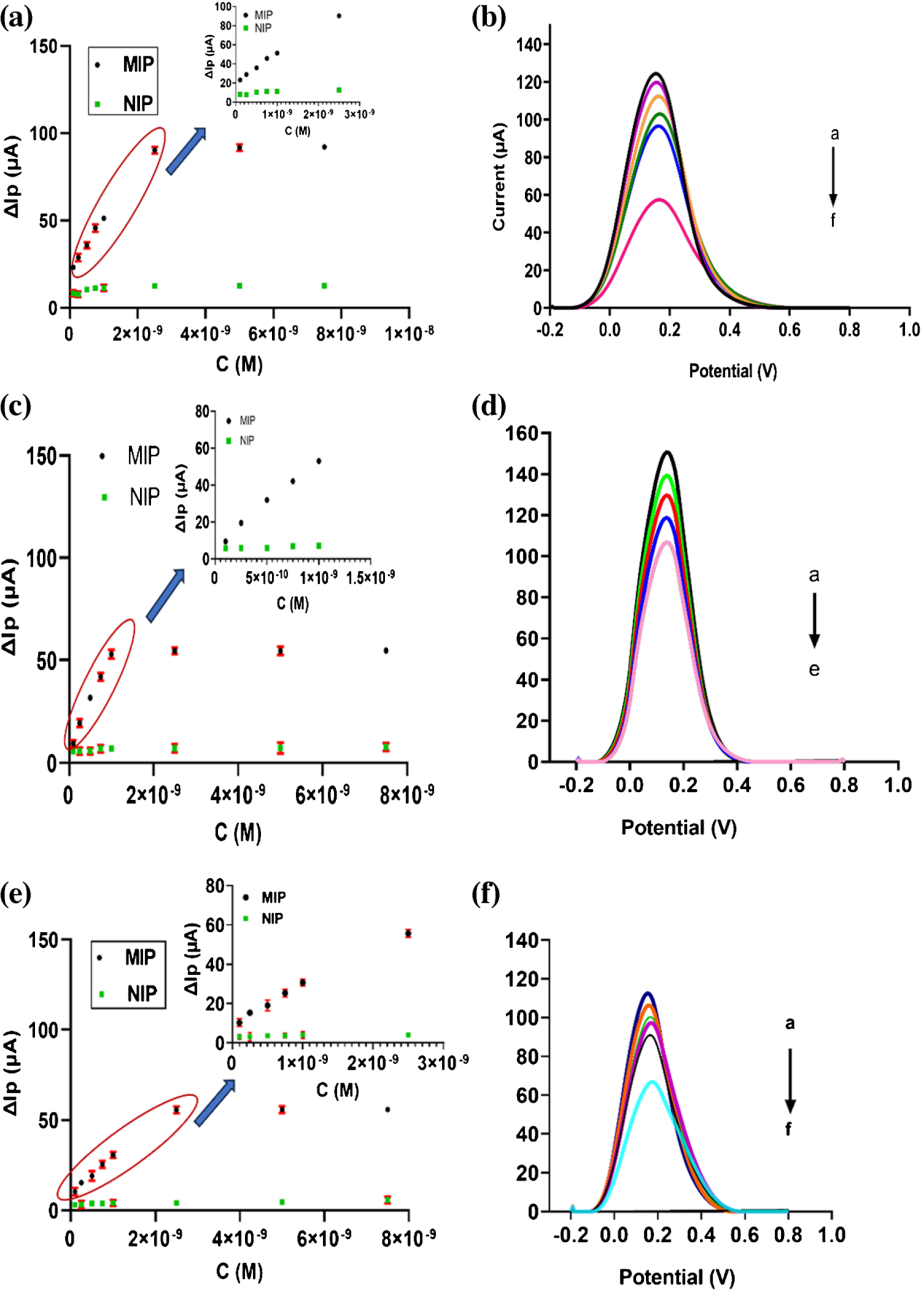
The linear calibration curves of 4-ABA@EMPA/MIP/GCE for EMPA determination for standard (**a**), plasma (**c**), urine (**e**); DPV voltammograms of redox probe solution after rebinding of different EMPA concentrations ranging between 0.1, 0.25, 0.50, 0.75, 1, and 2.5 nM for standard (**b**) and urine sample (**f**), 0.1 nM, 0.25 nM, 0.50 nM, 0.75 nM, and 1 nM for plasma sample (**d**). Conditions: for DPV, potential scan range −0.2 to +0.8V, scan rate 0.001587V s^−1^, step potential 8mV, modulation amplitude 50 mV, modulation time 0.05s, and interval time 0.5 s

Analytical validation and the performance of the 4-ABA@EMPA/MIP/GCE sensor were evaluated in the standard solution to determine EMPA. For this purpose, indirect measurements were performed by using the redox probe (5 mM [Fe(CN)_6_]^3−/4−^ solution) in the concentration range between 1.0 × 10^–10^ and 2.5 × 10^–9^ M. 4-ABA@EMPA/MIP/GCE sensor gave a linear response in this concentration range; however, the NIP-based sensor did not produce a logical response because it did not have specific cavities for EMPA (Fig. 4a).

The corresponding regression equation was found as Δ*I*_2_ (μΑ) = 2.76 × 10^10^ (μA/M) × C(M) + 20.48 (*r* = 0.998), and the related DPV voltammograms obtained after the increasing concentrations of EMPA are given in Fig. 4b. The equations in the ICH guidelines for the “Standard deviation of the response (s) and the slope of the obtained calibration curve (m)” method were used to calculate the limit of detection (LOD) and the limit of quantitation (LOQ) values as LOD = 3 × s/m and LOQ = 10 × s/m [31, 32]. LOD and LOQ values were found as 2.75 × 10^−11^ M and 9.18 × 10^−11^ M, respectively. Regression data of the calibration line for EMPA on 4-ABA@EMPA/MIP/GCE are given in Table 1. On the other hand, it was observed that the saturation region of the sensor was reached at lower and upper concentrations of this range and could not be included in the linear concentration range.

**Table 1:** Regression data of the calibration line for EMPA determination on 4-ABA@EMPA/MIP/GCE

	Standard solution	Plasma sample	Urine sample
Linearity range (M)	1.0 × 10^–10^–2.5 × 10^–9^	1.0 × 10^–10^–1.0 × 10^–9^	1.0 × 10^–10^–2.5 × 10^–9^
Slope (µA M^−1^)	2.76 × 10^10^	2.12 × 10^10^	1.85 × 10^10^
SE of slope (µA M^−1^)	1.09 × 10^9^	3.62 × 10^9^	7.53 × 10^8^
Intercept (µA)	20.48	27.28	10.37
SE of intercept (µA)	1.09	1.87	0.74
Correlation coefficient (*r*)	0.998	0.999	0.996
LOD (M)	2.75 × 10^−11^	2.11 × 10^−11^	2.36 × 10^−11^
LOQ (M)	9.18 × 10^−11^	7.04 × 10^−11^	7.87 × 10^−11^
Repeatability of peak current (RSD%)*	1.84	1.59	0.94
Reproducibility of peak current (RSD%)*	1.98	1.96	1.97

^*^Each value is the mean of three experiments

In 1994, two cult group members attacked a civilian by lethal nerve agent VX in Osaka, Japan. The victim died 10 days after the chemical attack. Six months later, serum samples of the victim were analyzed by mass spectrometry techniques, and serum EMPA concentration was estimated as 1.25 µg/mL [3]. A limited number of OPCW-designated laboratories that specialize in biomedical samples analyze either urine or blood samples for the absence or presence of OPNAs and low limit concentration of OPNA biomarkers including EMPA in body fluids is found around 1 ng/mL by using sophisticated laboratory techniques like high-resolution mass spectrometry and well-trained laboratory personnel [1, 6, 9]. Later, Hamelin et al. [33] developed an analytical method for quantifying EMPA in serum samples using hydrophilic interaction liquid chromatography and tandem mass spectrometry. The LOD for EMPA was reported as 0.50 ng/mL [33]. However, in our study, the LOD values of EMPA in plasma and urine samples were found to be 0.0026 × 10^−4^ ng/mL and 0.0029 × 10^−4^ ng/mL, respectively. Additionally, a response above the LOD was noted as indicative of VX exposure. Table 2 shows the comparison of 4-ABA@EMPA/MIP/GCE with other analytical studies for the determination of EMPA in biological and environmental samples in terms of method, linear range, LOD/LOQ values, sample, and recovery (%) [34–40]. Accordingly, it can be seen that our current study is the first electrochemical study and has lower LOD values and better sensitivity than other studies in the literature. Furthermore, compared to other studies, it is evaluated that this 4-ABA@EMPA/MIP/GCE sensor was applied to both plasma and urine samples, unlike other studies, and the recovery % values obtained are better. These aspects emphasize the advantage of the sensor.

**Table 2:** Comparison of 4-ABA@EMPA/MIP/GCE with other published analytical studies for the determination of EMPA in biological and environmental samples

Method	Linear range	LOD/LOQ	Tested sample	Recovery (%)	Ref.
IPD-IC	50 ng/mL–1 μg/mL	40 ng/mL	Serum	84.9	[34]
LC-MS/MS	1–200 ng/mL	160 pg/mL	Urine	99.89–103.37	[35]
GC-MS	NA	0.14 ng/mL	Urine	70–73	[36]
IC-MS/MS	28–1.000 ng/mL	12 ng/mL	Urine	81	[37]
HPLC-MS/MS	250–25.000 ng/mL (plants) 100–25.000 ng/mL (soil)	80 ng/mL (plants) 30 ng/mL (soil)	Plants and soil	NA	[38]
LC-MS/MS	10–100 ng/mL	5.35 ng/mL	Urine	NA	[39]
IC-MS/MS	50–1.000 ng/mL (urine)	50 ng/mL	Urine	NA	[40]
4-ABA@EMPA/MIP/GCE	1.0 × 10^–10^–2.5 × 10^–9^ M	2.75 × 10^−11^ M	Plasma and urine	99.86–101.08	Our study

*IPD-IC* indirect photometric detection ion chromatography, *LC-MS/MS* liquid chromatography tandem mass spectrometry, *GC-MS* gas chromatography–mass spectrometry, *IC-MS/MS* ion chromatography tandem mass spectrometry, *HPLC-MS/MS* high-performance liquid chromatography tandem mass spectrometry, *NA* not available

### Application of 4-ABA@EMPA/MIP/GCE sensor to human plasma and urine samples

Human plasma and urine samples were used to assess the applicability and accuracy of the 4-ABA@EMPA/MIP/GCE sensor in biological fluid media. The stock solutions of plasma and urine samples were prepared, as explained in “Materials and methods.” The concentration range at which the sensor responded linearly was evaluated for both samples. While this range was found to be 1.0 × 10^–10^ M and 1.0 × 10^–9^ M for plasma, it was found to be 1.0 × 10^–10^ M and 2.5 × 10^–9^ M for urine. Concentrations outside this range constitute the saturation region of the sensor for these two environments (Fig. 4c and d). LOD and LOQ values close to each other were acquired (2.11 × 10^−11^ M and 7.04 × 10^−11^ M for plasma; 2.36 × 10^−11^ M and 7.87 × 10^−11^ M for urine, respectively). Other related regression data are given in Table 1. Corresponding DPV voltammograms are shown in Fig. 4e and f.

Additionally, recovery experiments in plasma and urine samples were performed by spiking two known concentrations of EMPA to demonstrate the 4-ABA@EMPA/MIP/GCE sensor’s accuracy in these biological fluids. Significant recovery (%) and RSD (%) values were acquired, confirming the sensor’s accuracy (Table 3).

**Table 3:** Recovery experiment results for plasma and urine samples

	Plasma sample no. 1	Plasma sample no. 2	Urine sample no. 1	Urine sample no. 2
Sample concentration (M)	2.50 × 10^−10^	5.00 × 10^−10^	2.50 × 10^−10^	5.00 × 10^−10^
Spiked amount (M)	2.50 × 10^−10^	2.50 × 10^−10^	2.50 × 10^−10^	2.50 × 10^−10^
Found amount (M)*	5.03 × 10^−10^	7.58 × 10^−10^	4.98 × 10^−10^	7.60 × 10^−10^
Average recovery (%)*	100.62	101.08	99.86	101.30
RSD (%) of recovery	1.98	2.63	2.11	2.18
Bias (%)	+0.62	+1.08	−0.62	+1.30

^*^Each value is the mean of five experiments

### Selectivity study

#### Imprinting factor (IF)

Integrating MIPs with electrochemical sensors provides superior selectivity thanks to creating target-specific cavities on the GCE surface. This feature makes it possible to determine the target analyte even in complex samples and in the presence of other similarly structured compounds. When considered in terms of EMPA analysis, high affinity and selectivity against other metabolites with a similar structure to EMPA that can be found in biological fluids are very important. To evaluate the affinity and selectivity, IMPA (major hydrolysis metabolite of nerve agent sarin), n-BPA (internal standard of EMPA), and MPA were selected. As a result of the measurements, Δ*I*_2_ values were calculated corresponding to MIP and NIP. It was found that the IF′ values obtained were high (over 1), indicating the high affinity of the developed sensor to EMPA (Table 4) Due to the changes in structural similarities, approximately 1.5, 2.5, and 3.5 times selectivity was achieved in this study, compared to IMPA, n-BPA, and MPA, respectively.

**Table 4:** IF study results on 4-ABA@EMPA/MIP/GCE sensor

	MIP/GCE		NIP/GCE		
	Δ*I*_2_/µA	IF_(MIP)_	Δ*I*_2_/µA	IF_(NIP)_	IF′_(MIP/NIP)_
EMPA	35.86	-	10.49	-	-
IMPA	17.12	2.09	6.45	1.63	1.29
n-BPA	16.99	2.11	11.31	0.93	2.28
MPA	10.11	3.55	10.07	1.04	3.41

#### Interference agents

Interference studies were conducted to examine whether the presence of some compounds found in biological fluids or drugs used by individuals affects the determination of the target molecule. For this purpose, mixing interfering agents such as dopamine, ascorbic acid, paracetamol, and some ions was prepared at a concentration ten times higher than EMPA. Recovery studies were performed using the 1:10 molar ratio mixtures of interfering agents and EMPA, and the results are given in Table 5. Based on the results, it is seen that common interfering agents do not have a significant effect on the determination of EMPA.

**Table 5:** Effect of interfering agents on determining EMPA using 4-ABA@EMPA/MIP/GCE

Interfering agents	Recovery (%)	RSD (%)
Dopamine	99.50	1.82
Ascorbic acid	101.96	2.09
Uric acid	98.12	1.79
Paracetamol	97.50	2.07
K^+^	100.59	1.96
Cl^−^	100.35	1.77
Na^+^	99.47	1.85
SO_4_^2−^	99.47	1.85
Mg^2+^	100.35	1.77
NO_3_^−^	100.59	1.96

## Conclusion

The current sensor 4-ABA@EMPA/MIP/GCE utilizes sensitive electrochemical techniques, specifically through thermal polymerization processes. This newly developed sensor exhibited distinct recognition and binding properties toward EMPA molecules, delivering a range of exceptional features, including cost-effectiveness, long-term stability, reproducibility, linear ranges, and sensitivity. 4-ABA@EMPA/MIP/GCE exhibited low LOD and LOQ values in standard, plasma, and urine solutions. Furthermore, it displayed superior repeatability and reproducibility with biological samples and standard solutions.

Developed sensor platform 4-ABA@EMPA/MIP/GCE, which detects the presence of the target analyte EMPA in plasma or urine samples of suspected chemical casualties, can confirm the alleged exposure to lethal nerve agent VX early after a chemical terrorist attack. Until our research, the analysis of OPNA metabolites in authentic biomedical samples has been conducted by using mostly complex sample preparation methods and expensive high-resolution mass spectrometry techniques. 4-ABA@EMPA/MIP/GCE is developed as an effective POC diagnosis tool for the detection of VX exposure directly in human biomedical samples without any sample pretreatment and it is the first electrochemical sensor study in the literature with low detection limits, high selectivity, and sensitivity compared with recent instrumental analysis techniques. It provides a promising analytical strategy for future research which will enable the detection of biomarkers of other OPNAs like sarin, soman, or Novichok’s in human biological samples.

### Supplementary Information

Below is the link to the electronic supplementary material.

Supplementary file1 (DOCX 112 KB)
