# Supplementary material for: Development of a molecularly imprinted polymer-based electrochemical sensor for the selective detection of nerve agent VX metabolite ethyl methylphosphonic acid in human plasma and urine samples
Source: Anal Bioanal Chem. 2024 Jan 25;416(6):1505–15. doi: 10.1007/s00216-024-05155-6 (PMC10861733; doi:10.1007/s00216-024-05155-6)
Supplement: Supplementary file 1 — Supplementary file1 (DOCX 112 KB) [file 216_2024_5155_MOESM1_ESM.docx]

Analytical and Bioanalytical Chemistry

**Electronic Supplementary Material**

**Development of a Molecularly Imprinted Polymer Based Electrochemical Sensor for the Selective Detection of Nerve Agent VX metabolite Ethyl Methylphosphonic Acid in Human Plasma and Urine Samples**

Sermet Sezigen^1*^ S. Irem Kaya^2^, Nurgul K Bakirhan^2^, Sibel A Ozkan^3^

^1^ University of Health Sciences, Dept. of Medical CBRN Defense, Ankara, Turkiye

^2^ University of Health Sciences, Gülhane Fcaulty of Pharmacy, Department of Analytical Chemistry, Ankara, Turkiye

^3^ Ankara University, Faculty of Pharmacy, Department of Analytical Chemistry, Ankara, Turkiye

*Email corresponding author: sermet.sezigen@sbu.edu.tr

| **(a)** | 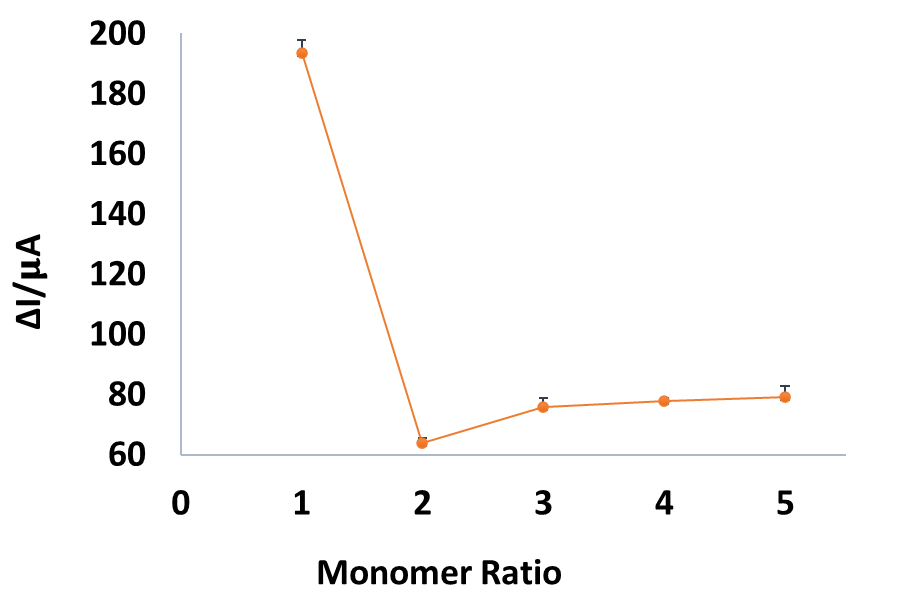 |
| --- | --- |
|  |  |
| **(b)** | 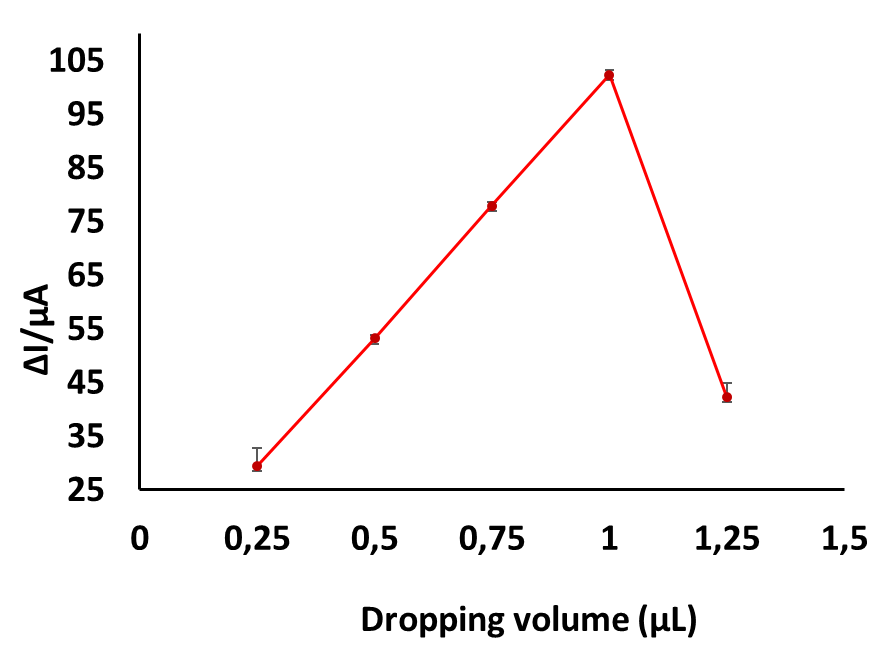 |
|  |  |
| **(c)** | 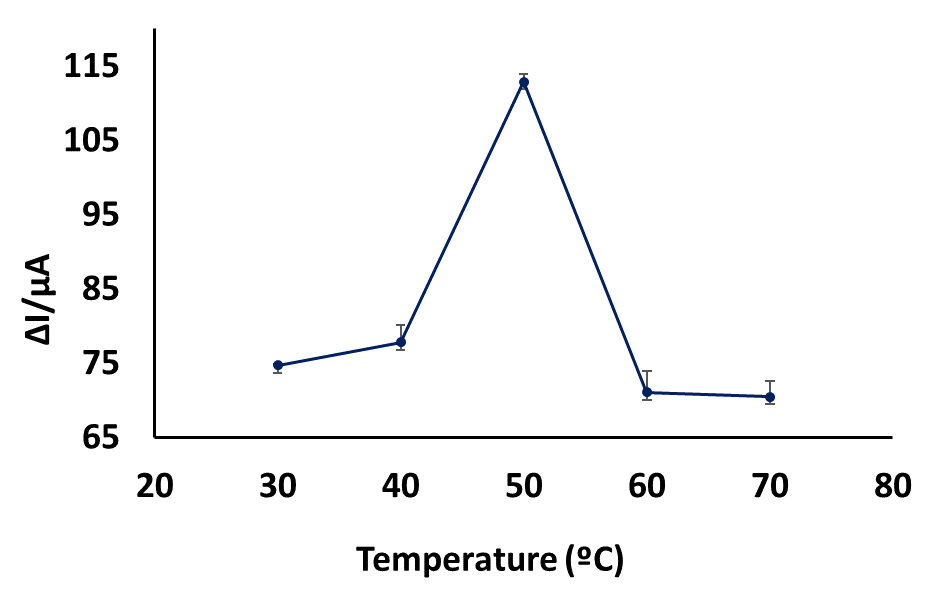 |
|  |  |
| **(d)** | 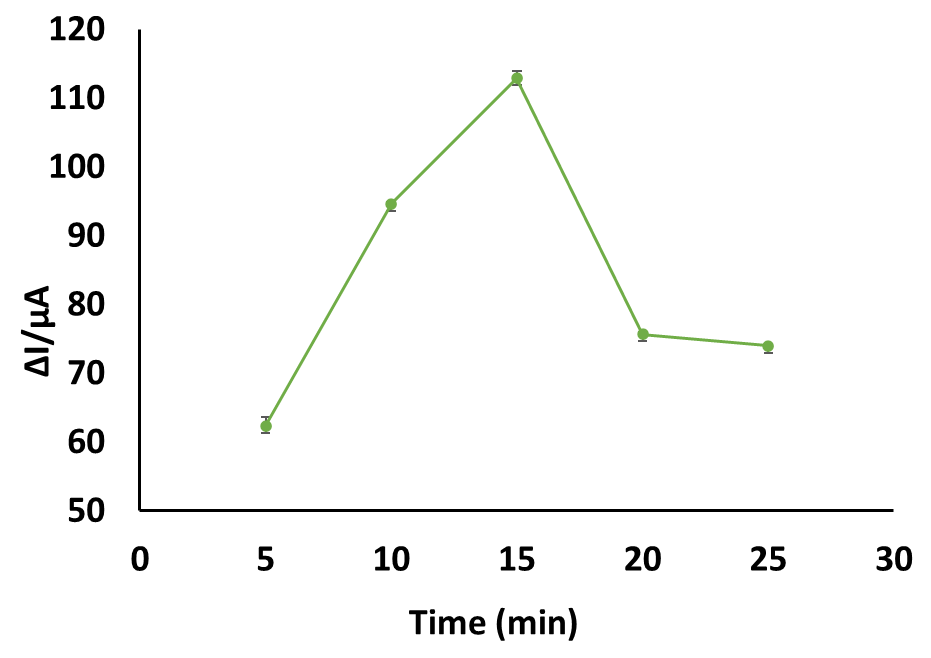 |
|  |  |
| **(e)** | 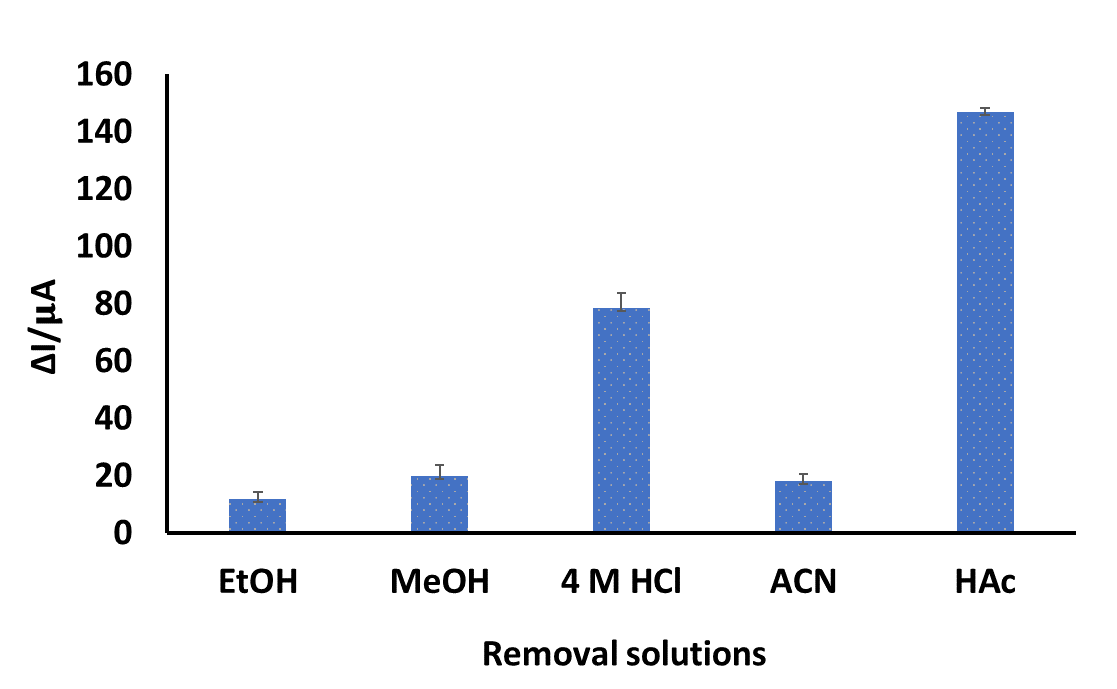 |
|  |  |
| **(f)** | 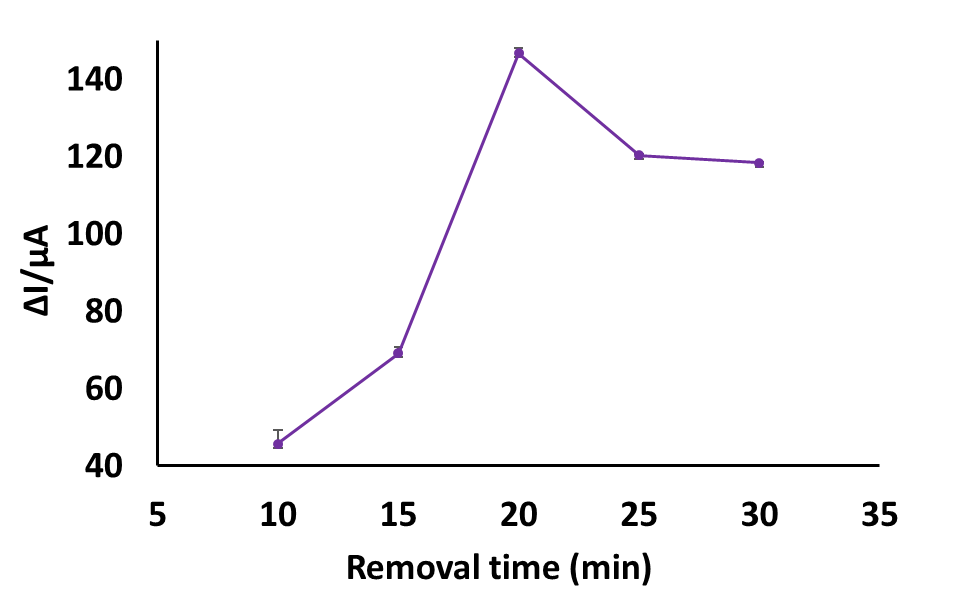 |
|  |  |
| **(g)** | 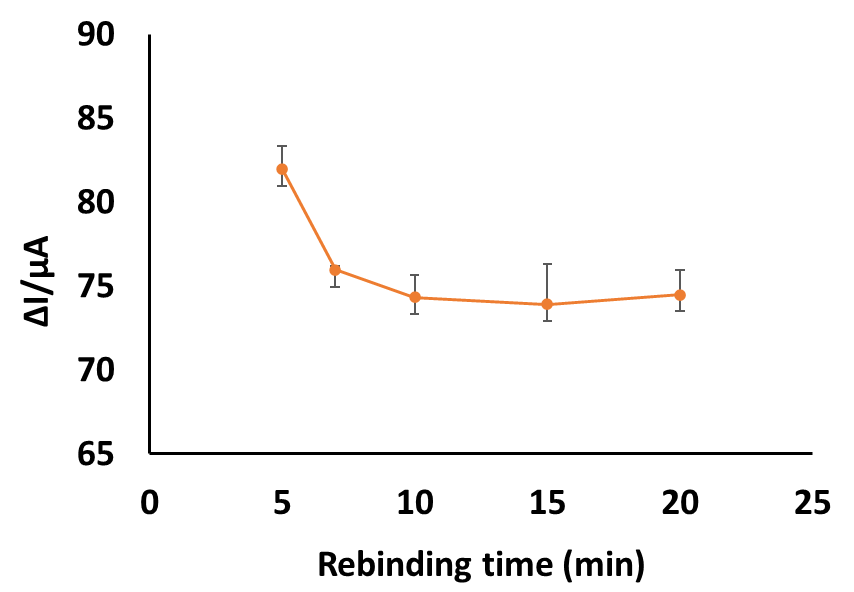 |

**Fig. S1** Plot of **(a)** monomer ratios versus ΔI values obtained after removal and after TP, **(b)** TP temperature, **(c)** TP time, **(d)** dropping volume, **(e)** different removal solutions, **(f)** different removal times versus ΔI values obtained after removal and after TP, **(g)** different rebinding times versus ΔI values obtained after removal and after rebinding of EMPA.
